# Laboratory and Field Evaluation of the Partec CyFlow MiniPOC for Absolute and Relative CD4 T-Cell Enumeration

**DOI:** 10.1371/journal.pone.0116663

**Published:** 2015-02-17

**Authors:** Djibril Wade, Papa Alassane Diaw, Géraldine Daneau, Abdoul Aziz Diallo, Souleymane Mboup, Tandakha Ndiaye Dieye, Luc Kestens

**Affiliations:** 1 Immunology unit, Laboratory of Bacteriology Virology, Le Dantec Hospital, Dakar, Senegal; 2 Laboratory of Immunology, Department of Biomedical Sciences, Institute of Tropical Medicine, Antwerp, Belgium; 3 Department of Biomedical Sciences, University of Antwerp, Antwerp, Belgium; Ghent University, BELGIUM

## Abstract

**Background:**

A new CD4 point-of-care instrument, the CyFlow miniPOC, which provides absolute and percentage CD4 T-cells, used for screening and monitoring of HIV-infected patients in resource-limited settings, was introduced recently. We assessed the performance of this novel instrument in a reference laboratory and in a field setting in Senegal.

**Methodology:**

A total of 321 blood samples were obtained from 297 adults and 24 children, all HIV-patients attending university hospitals in Dakar, or health centers in Ziguinchor. Samples were analyzed in parallel on CyFlow miniPOC, FACSCount CD4 and FACSCalibur to assess CyFlow miniPOC precision and accuracy.

**Results:**

At the reference lab, CyFlow miniPOC, compared to FACSCalibur, showed an absolute mean bias of -12.6 cells/mm^3^ and a corresponding relative mean bias of -2.3% for absolute CD4 counts. For CD4 percentages, the absolute mean bias was -0.1%. Compared to FACSCount CD4, the absolute and relative mean biases were -31.2 cells/mm^3^ and -4.7%, respectively, for CD4 counts, whereas the absolute mean bias for CD4 percentages was 1.3%. The CyFlow miniPOC was able to classify HIV-patients eligible for ART with a sensitivity of ≥ 95% at the different ART-initiation thresholds (200, 350 and 500 CD4 cells/mm3). In the field lab, the room temperature ranged from 30 to 35°C during the working hours. At those temperatures, the CyFlow miniPOC, compared to FACSCount CD4, had an absolute and relative mean bias of 7.6 cells/mm^3^ and 2.8%, respectively, for absolute CD4 counts, and an absolute mean bias of 0.4% for CD4 percentages. The CyFlow miniPOC showed sensitivity equal or greater than 94%.

**Conclusion:**

The CyFlow miniPOC showed high agreement with FACSCalibur and FACSCount CD4. The CyFlow miniPOC provides both reliable absolute CD4 counts and CD4 percentages even under the field conditions, and is suitable for monitoring HIV-infected patients in resource-limited settings.

## Introduction

CD4 T-cell enumeration is used together with HIV plasma viral load for monitoring HIV-infected patients. Viral load testing allows detecting and preventing early virologic failure during antiretroviral therapy (ART). However, the relative high cost and complexity of viral load assays limit their routine use in most middle and low income countries [1,2]. Therefore, CD4 counting is still the most commonly used assay to monitor persons suffering from HIV/AIDS in many settings. CD4 T-cell enumeration allows staging of HIV disease, deciding when to start treatment, and monitoring response to ART [1,3]. While Single Platform (SP) flow cytometry is conventionally considered as the gold standard for CD4 T-cell enumeration, standard flow cytometers are expensive to operate and maintain [4,5]. Moreover, they require highly trained personnel, an air conditioned lab and a cold chain to ship and store reagents, facilities which are not always available in resource-limited settings. To ensure decentralization of the HIV-monitoring services, more affordable CD4 dedicated flow cytometers have been introduced on the market, including FACSCount (BD Biosciences), CyFlow Counter (Partec), Guava EasyCD4 (Millipore), Apogee Auto40 (Apogee), PointCare NOW (PointCare) [5–13]. At the end of the previous decade, low-cost Point of Care (POC) technologies were being developed, mainly based on digital imaging [14–18]. Those CD4 POC technologies would help to facilitate early ART initiation and to reduce the proportion of patients lost to follow up [19–21]. The Pima CD4 (Alere), the only POC system actually on the market and which has been evaluated extensively, uses thermo-resistant disposable cartridges preloaded with lyophilized monoclonal antibodies (mAb) [18,22–25]. Although the Pima CD4 provides absolute CD4 T-lymphocyte counts in about 20 minutes after blood collection, it cannot provide a CD4 percentage (CD4%) which is essential for monitoring of pediatric patients [24,26–28]. Recently, Partec introduced a small handheld and portable flow cytometry-based CD4 POC, the CyFlow miniPOC (Partec GmbH, Munster, Germany) which provides absolute and percentage CD4 T-cells. It is a closed system operating with Partec miniPOC CD4% count kit—dry, which eliminates the need for cold chain [2,29]. The kit is intended for 20 tests and includes ready-prepared sample tubes (yellow lid) containing lyophilized CD4 PE and CD45 PE-Dy647 mAb, tubes with lyophilized Count Check Beads green (blue lid), buffer tubes (orange lid for buffer 1, black lid for buffer 2, and blue lid for dilution of Count Check beads), and sheath fluid. The CyFlow miniPOC runs on regular power supplies. The company claims it runs optionally on a rechargeable battery pack or solar panels, and it can run up to 250 tests per day. Therefore, the CyFlow miniPOC would be an interesting candidate for CD4 T-cell counting in adult and pediatric HIV-infected patients in low-income countries. We evaluated the performance of the CyFlow miniPOC in the National Reference Laboratory in Dakar and in a peripheral laboratory, and compared results with those obtained from FACSCalibur using Trucount tubes, and from FACSCount for both absolute CD4 counts and CD4%.

## Methodology

### Ethics statement

We used the excess of routine blood samples from HIV-infected patients attending the clinic for routine CD4 T-cell enumeration. Blood sample records were directly anonymized and de-identified by giving them a consecutive study code prior to analysis. The study was approved by the Institutional Review Board of the Institute of Tropical Medicine, Antwerp (Belgium) and the National Ethical Committee of the Ministry of Health (Senegal) that waived the need of informed consent. For the purpose of this independent study, the CyFlow miniPOC device and its reagent kits, the FACSCount CD4 reagents, the Trucount tubes and the monoclonal antibodies were purchased.

### Study design and participants

This study was conducted in two phases, the first one in the reference laboratory at the Immunology unit of Le Dantec hospital Dakar (March—May 2014), and the second in a peripheral laboratory at the Silence health center in Ziguinchor at 450 Km from Dakar (end May—June 2014). Blood samples, collected into K3-EDTA tubes, were taken consecutively from HIV-patients attending the clinic for routine CD4 counting.

The phase I consisted in evaluating the CyFlow miniPOC performances in a controlled laboratory environment where FACSCalibur and FACSCount CD4 were set as reference instruments.

In phase II, the CyFlow miniPOC was installed in a room without air conditioning, and results were compared with those from FACSCount CD4, which was used as reference instrument. During the study, working temperature in that room ranged from 30°C to 35°C. The FACSCount instrument was installed in a room with air-conditioning, as recommended.

### Precision assessment

Intra-assay and inter-assay variations were assessed on 3 blood samples with clinically relevant CD4 counts (< 200, 300–500, > 500 cells/mm^3^). The intra-assay, which assesses the tube-to-tube variability, including errors due to operator, was determined by repeating 10 times the entire CD4 staining procedure on the 3 blood samples. Inter-assay variation (day-to-day) was assessed by analysing each sample at three time points after blood collection (within 6h, 24h and 48h) on blood stored at room temperature. The percentage coefficient of variation (%CV) was calculated for intra-assay and inter-assay [22]. The sample carry over was measured by analyzing three sample pairs, each pair with one sample with a CD4 count greater than 600 cells/mm^3^ read in duplicate (recorded as a_l_ and a_2_) followed by a second sample with a CD4 count lower than 300 cells/mm^3^ read in duplicate too (recorded as b_1_ and b_2_), and the Broughton coefficient (k) was calculated [23,30]. The values of the internal quality control Count Check beads run daily on the CyFlow miniPOC were used to calculate the %CV of instrument precision.

### Agreement between methods

CD4 counts were measured in whole blood samples within 6 hours after venipuncture on: FACSCalibur, FACSCount CD4, and CyFlow miniPOC. All measurements were performed according to manufacturer’s instructions by different operators to allow a blind reading. Before running samples, internal quality control beads were successfully tested daily on CyFlow miniPOC (Partec Count Check), on FACSCount (BD FACSCount Control), and on FACSCalibur (BD Calibrite).

The CyFlow miniPOC, flow cytometry-based CD4 POC, provides absolute and percentage CD4 T-cells. Briefly, 20mm^3^ of whole blood were added into a sample tube (on top of the lyophilized antibody spot at the bottom of the tube) gently mixed and incubated for 15min (mix again after 5min) in the dark at room temperature. Then, Buffer 1 was poured and mixed, and the Buffer 2 was added directly prior to the measurement on the CyFlow miniPOC. Stained blood was completely aspirated with a new syringe until the plunger reaches the position 1 ml (avoid having air bubble in the extremity of the syringe). The syringe was attached to the device, analysis started and the results (CD4 count and CD4%) were available in less than 2min.

For FACSCalibur and FACSCount CD4 reference methods, samples were prepared, as previously described, to provide both absolute and percentage of CD4 T-cells. A CD3/CD4/CD45 panel in Trucount tubes was used on FACSCalibur, as previously described [4,12,23]. The FACSCalibur and the FACSCount CD4 are monitored by external quality control programs QASI (Quality Assessment and Standardization for Immunological measures relevant to HIV/AIDS) and UK NEQAS (United Kingdom National External Quality Assessment Service).

### Statistical analysis

The concordance correlation coefficient (ρ_c_ = ρ x C_b_) assesses the degree to which pairs of observations fall on the 45° line through the origin [31]. Passing-Bablok regression was used for method comparison [32]. Pollock and Bland Altman analyses were used to assess relative and absolute mean bias, respectively between absolute CD4 counts and CD4 percentages obtained by both methods. Mean bias (or trueness) and limits of agreement (LOA = mean bias ± 1.96 x SD) were calculated [33,34]. The percentage similarity between the results obtained from 2 different instruments was calculated for each sample. For each group, the mean similarity and the relative standard deviation (RSD) were determined [22,35]. Sensitivity and specificity to identify patients eligible for ART were calculated at commonly used CD4 thresholds (200, 350 and 500 cells/mm^3^) to start ART. FACSCalibur or FACSCount CD4 results (CD4 count & CD4%) were set as the reference to determine ART eligible patients, and calculate misclassification, sensitivity and specificity.

## Results

### Study population

A total of 188 HIV-infected patients were included in the first part of this study, of which, 35% (65/188) were male, and 64% (120/188) received ART. There were 169 adults and 19 children, including 5 under 5 years, with a global median (range) age of 39 (1–68) years. They had a median (range) CD4 count of 334 (10–1508) cells/mm^3^, and 51 had a low CD4 count ≤ 200, 87 had a CD4 count between 201 and 500, and 50 subjects had a CD4 count > 500.

At the Silence health center (second phase), 133 HIV-infected patients were enrolled, of which, 20% (26/133) were male, and 60% (80/133) received ART. There were 128 adults and 5 children, including only 1 under 5 years, with a global median (range) age of 43 (3–64) years. They had a median CD4 count (range) of 337 (2–1024) cells/mm^3^, and 35 of them had a CD4 count ≤ 200, 58 had a CD4 count between 201 and 500, and 40 had a CD4 count > 500.

### Precision assessment

Table 1 summarizes the precision (%CVs) of the CyFlow miniPOC on replicates from samples with low, medium and high CD4 count. The CyFlow miniPOC showed a mean intra-assay variation (%CV) of 3.6% for CD4 counts and 4.5% for CD4%. The mean inter-assay %CV was 6.1% for CD4 counts and 3.2% for CD4%. Values at 48h did not seem inferior to values at 24h, and only the sample with a high CD4 count showed a drop from 1153 cells/mm^3^ at day 0 to 906 cells/mm^3^ at day 1, leading to the high CV of 13.45%. There was no significant carry over with a mean k of 0.12% for CD4 count, and 0.00% for CD4%. Based on the values obtained daily from the readings of the Count Check control beads, the CyFlow Counter showed a mean instrument precision of 2.8%.

**Table 1:** Variability of the CyFlow miniPOC.

		**Low**	**Medium**	**High**
Intra-assay (n = 10)	Mean CD4 count (cells/mm^3^)	133	346	660
	CV for CD4 count	3.94%	4.88%	2.09%
	CV for CD4%	6.06%	4.74%	2.67%
Inter-assay (n = 3)	Mean CD4 count (cells/mm^3^)	111	331	999
	CV for CD4 count	2.27%	2.57%	13.45%
	CV for CD4%	6.19%	0.00%	3.45%

**n** refers to the number of replicates

**CV** means coefficient of variation

**Low** means CD4 < 200 cells/mm^3^

**Medium** means CD4 between 300–500 cells/mm^3^

**High** means CD4 > 500 cells/mm^3^.

### Agreement between methods

**Fig 1:**
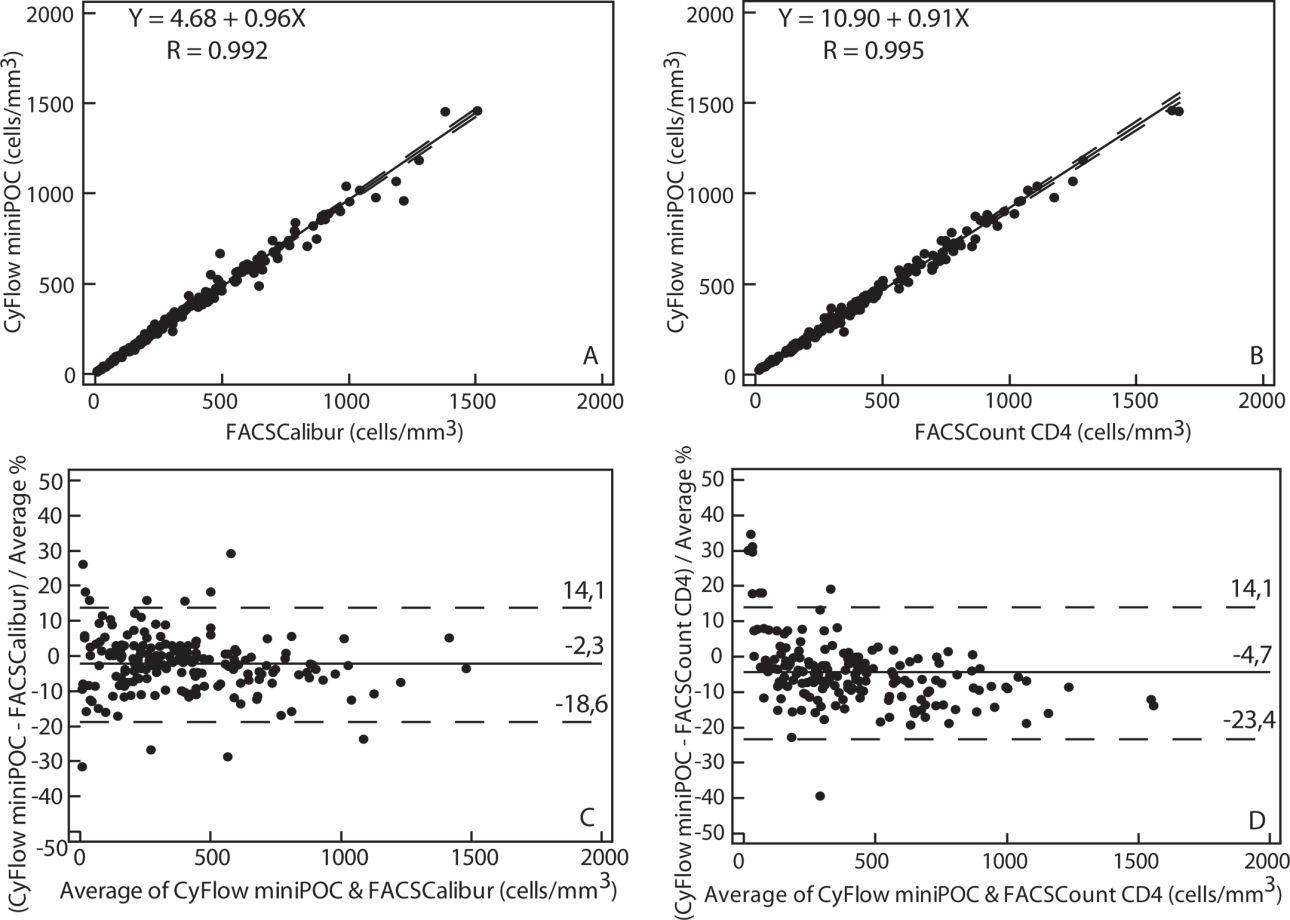
Comparison between CyFlow miniPOC and conventional flow cytometers FACSCalibur and FACSCount CD4: absolute CD4 counts obtained by CyFlow miniPOC were compared to those from FACSCalibur (A and C) and those from FACSCount CD4 (B and D) using Passing-Bablok and Pollock analyses, respectively. In Passing–Bablok regression plots, the solid line represents the regression line and the dashed lines represent the 95% confidence interval for the regression line. In Pollock plots, the solid line represents the mean bias. The dashed lines represent the mean bias ± 1.96 SD, which are the upper and lower limits of agreement (LOA).

**Phase I: Evaluation of the CyFlow miniPOC performances in a controlled laboratory environment.** Fig. 1 shows absolute CD4 count comparisons between CyFlow miniPOC and the conventional flow cytometers (FCM) in the reference laboratory.

Comparison between CyFlow miniPOC and FACSCalibur showed a mean absolute bias (LOA) of -12.6 (-91.6–66.3) cells/mm^3^, a mean relative bias (LOA) of -2.3% (-18.6–14.1), and a mean similarity (RSD) of 99% (6%). According to FACSCalibur results, the CyFlow miniPOC misclassified 2% (4/185), 1% (2/185) and 3% (5/185) of patients, respectively at the thresholds of 200, 350 and 500 cells/mm^3^ used for ART-initiation. The respective values of sensitivity were 96% (48/50), 99% (95/96) and 97% (131/135), and those of specificity were 99% (132/134), 99% (88/89) and 98% (49/50). Among the misclassified patients, the maximum bias observed was 25 cells/mm^3^ at the threshold of 200 cells/mm^3^, and 10 cells/mm^3^ at the threshold of 350 cells/mm^3^. At the threshold of 500 cells/mm^3^, the maximum bias was 168 cells/mm^3^ (Table 2).

**Table 2:** CD4 value for samples around the thresholds misclassified by the CyFlow miniPOC according to the FACSCalibur reference.

**Thresholds**	**FACSCalibur (cells/mm^3^)**	**FACSCount CD4 (cells/mm^3^)**	**CyFlow miniPOC (cells/mm^3^)**	**Bias (cells/mm^3^)**
**200 cells/mm** ^**3**^	**197**	213	222	+25
	**200**	224	215	+15
	**205**	196	183	-22
	**211**	206	196	-15
**350 cells/mm** ^**3**^	**347**	367	357	+10
	**354**	368	348	-6
**500 cells/mm** ^**3**^	**483**	586	523	+40
	**487**	504	517	+30
	**495**	667	663	+168
	**501**	486	495	-6
	**502**	467	458	-44

Each line corresponds to one patient. **Bias** refers to the difference between CyFlow miniPOC and FACSCalibur results.

Comparison between CyFlow miniPOC and FACSCount CD4 showed a mean absolute bias (LOA) of -31.2 (-119.9–57.4), a mean relative bias (LOA) of -4.7% (-23.4–14.1), and a mean similarity (RSD) of 98% (5%). According to FACSCount CD4 results, the CyFlow miniPOC misclassified 1% (2/181), 4% (7/181) and 1% (2/181) of patients, and showed sensitivity of 100% (46/46), 98% (89/91) and 100% (126/126), and specificity of 99% (133/135), 94% (85/90) and 96% (53/55), respectively at the thresholds of 200, 350 and 500 cells/mm^3^.

Finally, the comparison between FACSCount CD4 and FACSCalibur, the two CD4 reference instruments in this study, showed a coefficient correlation R of 0.990 (*y* = -4.76 + 1.05*x*), a mean absolute bias (LOA) of 17.9 (-73.8–109.6), a mean relative bias (LOA) of 2.3% (-19.7–24.8) and a mean similarity (RSD) of 100% (8%).

**Fig 2:**
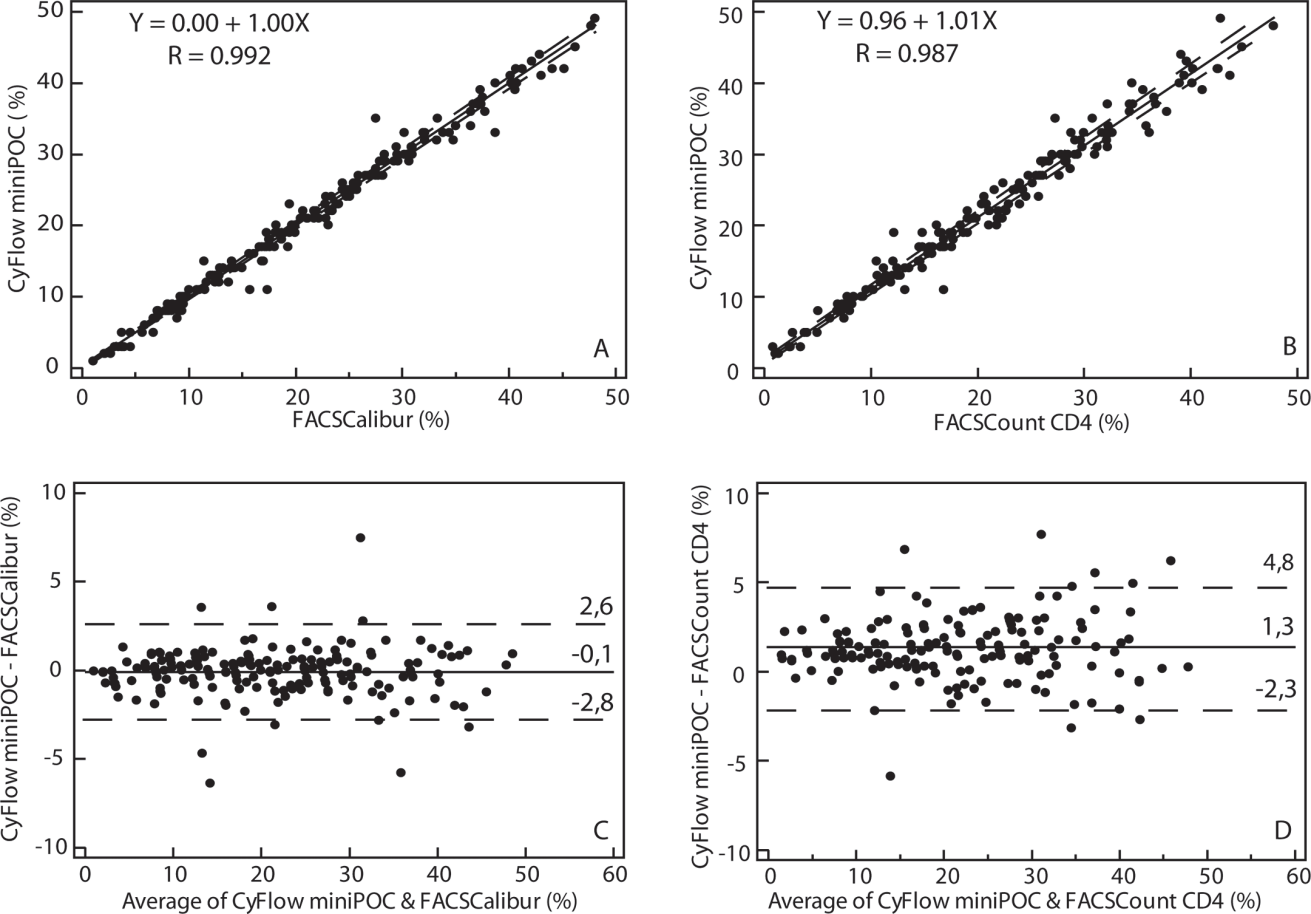
Comparison between CyFlow miniPOC and FACSCalibur and FACSCount CD4. CD4% obtained by CyFlow miniPOC were compared to those from FACSCalibur (A and C) and those from FACSCount CD4 (B and D), using Passing-Bablok and Bland-Altman analyses respectively. In Passing–Bablok plots, the solid line represents the regression line and the dashed lines represent the 95% confidence interval for the regression line. In Bland–Altman plots, the solid line represents the mean bias. The dashed lines represent the mean bias 6 1.96 SD, which are the upper and lower LOA.

Fig. 2 illustrates CD4% comparisons between CyFlow miniPOC and the conventional FCM in the reference laboratory.

Comparison between CyFlow miniPOC and FACSCalibur showed a mean absolute bias (LOA) of -0.1% (-2.8–2.6), and a mean similarity (RSD) of 99% (7%).

Comparison between CyFlow miniPOC and FACSCount CD4 showed a mean absolute bias (LOA) of 1.3% (-2.3–4.8), and a mean similarity (RSD) of 105% (12%).

The FACSCount CD4 and FACSCalibur comparison showed an R of 0.991 (*y* = -1.10 + 0.99*x*), a mean absolute bias (LOA) of -1.3% (-4.3–1.6), and a mean similarity (RSD) of 96% (6%).

The agreement between CyFlow miniPOC and each of the two reference systems (FACSCalibur and FACSCount CD4) was similar to the agreement between FACSCalibur and FACSCount CD4.

Table 3 summarizes the detailed comparisons between CyFlow miniPOC and FACSCalibur in the reference lab, within the CD4 categories. Table 4 summarizes the detailed comparisons between CyFlow miniPOC and FACSCount CD4 in the reference lab, within the CD4 categories.

**Table 3:** Comparison between CyFlow miniPOC and FACSCalibur in the reference laboratory.

	**All**	**CD4 ≤ 200**	**200 < CD4 ≤ 500**	**CD4 > 500**
**N**	**188**	**51**	**87**	**50**
**Concordance correlation coefficient (ρ_c_)**				
CD4 count	0.989	0.988	0.948	0.950
CD4%	0.992	0.985	0.982	0.986
**Absolute or relative mean bias (LOA)**				
CD4 count	-12.6 UI (-92–66)	-1.1 UI (-19–17)	-1.9 UI (-61–57)	-42.5 UI (-154–69)
CD4 count	-2.3% (-19–14)	-1.3% (-22–19)	-0.9% (-16–14)	-5.5% (-19–8)
CD4%	-0.1% (-3–3)	-0.1% (-2–2)	-0.1% (-3–3)	-0.2% (-3–3)
**Similarity: mean (RSD)**				
CD4 count	99% (6%)	100% (5%)	100% (8%)	97% (3%)
CD4%	99% (7%)	98% (9%)	99% (8%)	100% (2%)

**N** means number of samples

**LOA** means limits of agreement

**RSD** means relative standard deviation

UI = cells/mm^3^.

**Table 4:** Comparison between CyFlow miniPOC and FACSCount CD4 in the reference laboratory, Immunology unit, LBV Dakar.

	**All**	**CD4 ≤ 200**	**200 < CD4 ≤ 500**	**CD4 > 500**
**N**	**188**	**51**	**87**	**50**
**Concordance correlation coefficient (ρ_c_)**				
CD4 count	0.983	0.979	0.951	0.923
CD4%	0.980	0.941	0.958	0.964
**Absolute or relative mean bias (LOA)**				
CD4 count	-31.2 UI (-120–57)	-1.9 UI (-24–20)	-18.6 UI (-68–31)	-76.8 UI (-184–30)
CD4 count	-4.7% (-23–14)	2.2% (-23–27)	-5.3% (-20–9)	-9.4% (-21–2)
CD4%	1.3% (-2–5)	1.4% (-1–3)	1.3% (-3–5)	1.1% (-2–5)
**Similarity: mean (RSD)**				
CD4 count	98% (5%)	102% (7%)	98% (4%)	96% (3%)
CD4%	105% (12%)	115% (23%)	103% (5%)	102% (3%)

**N** means number of samples

**LOA** means limits of agreement

**RSD** means relative standard deviation

UI = cells/mm^3^.

**Fig 3:**
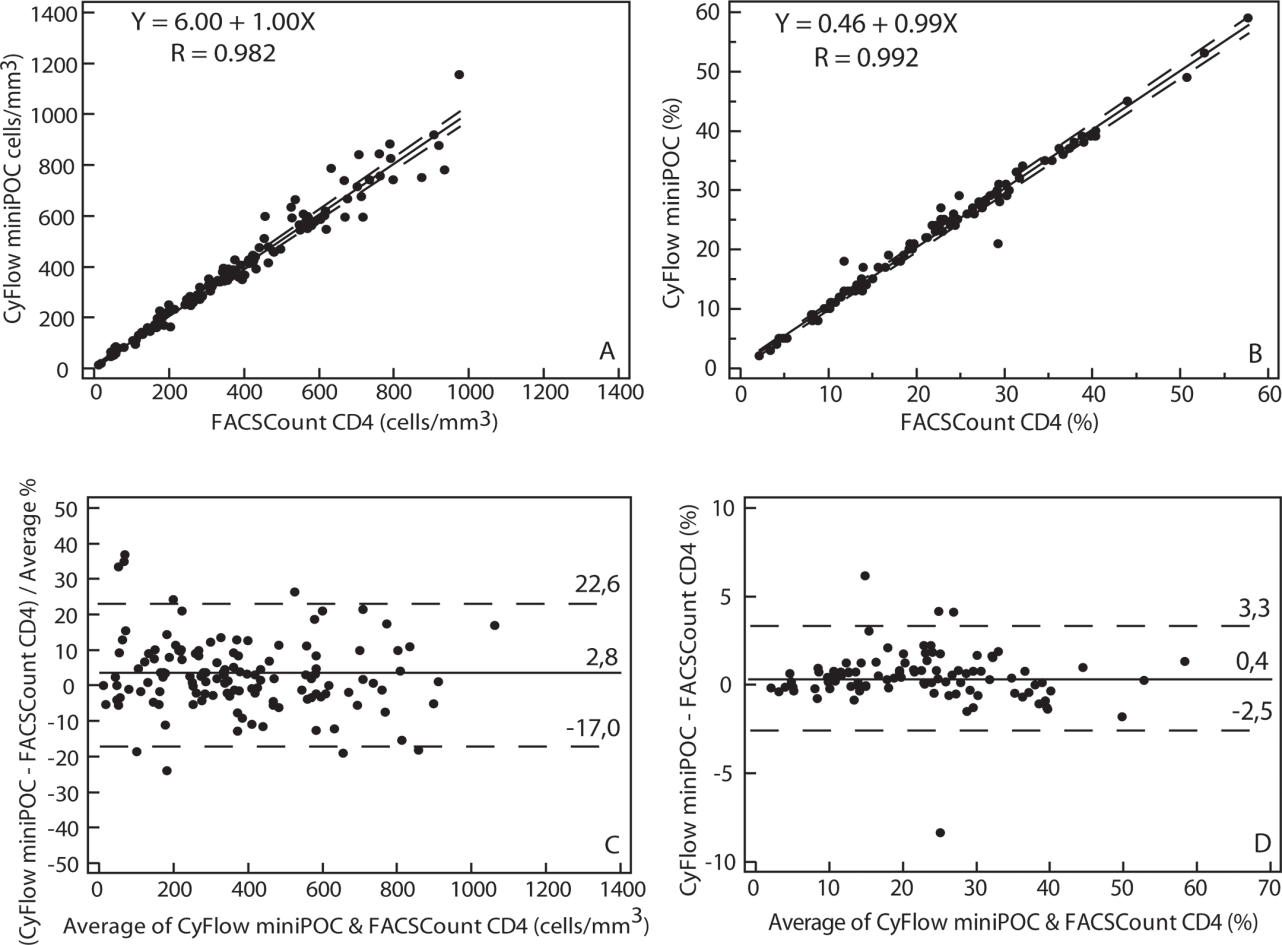
Comparison between CyFlow miniPOC and FACSCount CD4 in field conditions: CD4 counts from both instruments were analyzed using Passing-Bablok (A) and Pollock (C). CD4% from both instruments were analyzed by Passing-Bablok (C) and Bland-Altman (D). In Passing–Bablok plots, the solid line represents the regression line and the dashed lines represent the 95% confidence interval for the regression line. In Pollock and Bland–Altman plots, the solid line represents the mean bias. The dashed lines represent the mean bias 6 1.96 SD, which are the upper and lower LOA.

**Phase II: field validation of the CyFlow miniPOC at Silence health center.** Fig. 3 depicts the comparison between CyFlow miniPOC and FACSCount CD4.

For absolute CD4 counts, CyFlow miniPOC showed a mean absolute bias (LOA) of 7.6 (-80.5–95.8), a mean relative bias (LOA) of 2.8% (-17.0–22.6), and a mean similarity (RSD) of 102% (5%). Using FACSCount CD4 results as reference for patients classification, the CyFlow miniPOC misclassified 2% (3/129), 4% (5/129) and 2% (2/129) of patients, and showed sensitivity of 94% (33/35), 96% (65/68) and 98% (92/94), and specificity of 99% (93/94), 97% (59/61) and 100% (35/35) respectively at the different CD4-based treatment initiation thresholds of 200, 350 and 500 cells/mm^3^.

For CD4 percentages, CyFlow miniPOC showed a mean absolute bias (LOA) of 0.4% (-2.5–3.3), and a mean similarity (RSD) of 101% (4%).

Comparisons between CyFlow miniPOC and FACSCount CD4 in the field within the CD4 categories are detailed in Table 5. Comparisons between CyFlow miniPOC and FACSCount CD4 in the reference lab and in the field showed a statistically different bias (p < 0.05) in overall data and within each of the CD4 categories. The CyFlow miniPOC showed better performance on the CD4 percentages in Ziguinchor compared to Dakar. However, on absolute CD4 counts, the performance was not in favor of one site (Tables 4 & 5).

**Table 5:** Comparison between CyFlow miniPOC and FACSCount CD4 in the field setting, Silence health center, Ziguinchor.

	**All**	**CD4 ≤ 200**	**200 < CD4 ≤ 500**	**CD4 > 500**
**N**	**133**	**35**	**58**	**40**
**Concordance correlation coefficient (ρ_c_)**				
CD4 count	0.981	0.980	0.954	0.856
CD4%	0.991	0.992	0.972	0.985
**Absolute or relative mean bias (LOA)**				
CD4 count	7.6 UI (-81–96)	4.6 UI (-17–26)	6.2 UI (-41–53)	12.3 UI (-137–162)
CD4 count	2.8% (-17–23)	4.8% (-19–29)	2.4% (-14–18)	1.9% (-20–23)
CD4%	0.4% (-3–3)	0.1% (-1–2)	0.5% (-3–4)	0.4% (-3–3)
**Similarity: mean (RSD)**				
CD4 count	102% (5%)	103% (7%)	101% (4%)	101% (6%)
CD4%	101% (4%)	99% (3%)	102% (3%)	101% (5%)

**N** means number of samples

**LOA** means limits of agreement

**RSD** means relative standard deviation

UI = cells/mm^3^.

The CyFlow miniPOC failed to analyze 3 samples in the reference lab, while 5 and 4 errors were reported on FACSCount CD4 respectively in the reference lab and in the field lab.

## Discussion

The recent introduction of CD4 Point-of-care technologies has contributed to widen access to ART in resource-limited settings. Many POCs are much more affordable than the conventional flow cytometry based instruments and better adapted to the field as they are very mobile, easy to use and do not require a cold chain to store reagents [2,16,36]. In the present study, we independently evaluated the CyFlow miniPOC, a relatively new flow cytometry-based POC. Our study is the first to report on the performance of CyFlow miniPOC. The CyFlow miniPOC system was compared to two conventional flow cytometers, FACSCount CD4 and FACSCalibur.

The CyFlow miniPOC showed good precision with %CV < 7%, similar to precisions reported on other CD4 dedicated flow cytometers [6–8,10,22,23,37–43]. The high %CV (13.45%) of inter assay variability for the high CD4 count specimen (Table 1) can be explained by the CD4 count drop from 1153 cells/mm^3^ on fresh blood (within 6h) to 906 cells/mm^3^ at 24h after venipuncture. This variability was not inherent to the CyFlow miniPOC, but to the sample itself. Indeed, when we checked on FACSCalibur for that sample at day 1, the CD4 result was similar. The CyFlow miniPOC seems to be more precise than the Pima CD4 using venous blood, which indeed showed a higher %CV, superior to 10% [21,22,44]. The CD4 counts and CD4% obtained from the CyFlow miniPOC showed high correlation and small bias as compared to the conventional flow cytometers FACSCalibur (-12.6 cells/mm^3^) and FACSCount CD4 (-31.2 cells/mm^3^ in reference lab, and 7.6 cells/mm^3^ in the field). The agreement between CyFlow miniPOC and these conventional flow cytometers is similar to the agreement between FACSCount CD4 and FACSCalibur. These findings are similar to those reported in previous studies on other CyFlow instruments [38,39,45]. The CyFlow miniPOC bias is similar to those reported on other POC systems: MBio CD4 and Pima CD4 [16,18,21–24,28] while other studies reported higher bias on Pima CD4 [21,27,44,46]. In the reference laboratory, the mean bias (absolute and relative) increased with the CD4 count. This finding is similar to the findings reported on CyFlow Counter [22] and on Pima CD4 [21–23,27]. In the field, there was an increase in absolute bias and a decrease in relative bias. Although the performances (biases) between the reference and the field sites were statistically different, this was not clearly in favor of one of the two sites (some parameters better in Dakar, some in Ziguinchor), and the CyFlow miniPOC showed good agreement with FACSCount CD4 in both sites.

The CyFlow miniPOC showed low rates of misclassification of patients (< 5%) at the different CD4 treatment thresholds which are similar to those reported for other dedicated flow cytometers [8–10,22,43,45,47]. At the threshold of 200 cells/mm^3^, which would have more critical consequences if a patient is misclassified upward (no treatment provided), the CyFlow miniPOC showed a rate of misclassification ≤ 2% with only 2 patients who would have their ART-initiation postponed, and for only a short delay (maximum bias of 25 cells/mm^3^). At the threshold of 350 cells/mm^3^, only 3 (2%) patients would have their ART-initiation postponed with a maximum bias of 63 cells/mm^3^ according to FACSCount CD4. At the threshold of 500 cells/mm^3^, among the 4 (2%) patients who would have their ART-initiation postponed, only one patient showed a bias of 168 cells/mm^3^ when relying on CyFlow miniPOC rather than on the FACSCalibur. However, the CyFlow miniPOC and the FACSCount CD4 provided similar results for that sample (663 and 667 respectively) suggesting that the bias might be attributable to the FACSCalibur (495 cells/mm^3^), possibly as a result of a pipetting error. Fortunately, most of misclassified patients by the CyFlow miniPOC would benefit to an early ART-initiation which helps to reduce morbidity and consequently HIV-transmission [48] and due to their small number would be of low importance for ART stock consumption.

With an incubation time of 15min, and a possibility to prepare samples individually or in batches, and using less than 2min per sample reading, the CyFlow miniPOC can easily run a batch of 10 samples per hour. However, the analyses cannot be automated. The CyFlow miniPOC has a turnaround time of 20min similar to other POCs. This is an important asset and limits the number of patients lost to follow up as clinicians can take adequate treatment decisions immediately.

To ensure reliability of results, the FACSCalibur and FACSCount CD4, used in this study, present good scores in the external quality assessment programs such as QASI and UK NEQAS. In addition, the operators received appropriate training on the use of the CyFlow miniPOC which took 3h (one morning), and daily calibration (internal quality control) was performed daily on FACSCalibur, FACSCount and CyFlow miniPOC before running samples.

The fact that our evaluation took place in two different settings reinforces the strength of our results, but also shows that results can be slightly different due to the type of setting (in our case, the operator was the same), and that any evaluation should be done in both settings.

In conclusion, the CyFlow miniPOC provides reliable absolute CD4 counts and CD4 percentages, which are in excellent agreement with those obtained from the conventional flow cytometers. With a batch of 10 samples per hour, a technician could easily analyze up to 60 samples/day using the CyFlow miniPOC. Thus, the CyFlow miniPOC is suitable for monitoring of HIV-infected patients and can be used in a central or peripheral laboratory. Further studies are required for a definitive performance statement.
